# Barriers to the National Onchocerciasis Control Programme at operational level in Cameroon: a qualitative assessment of stakeholders’ views

**DOI:** 10.1186/s13071-019-3497-5

**Published:** 2019-05-20

**Authors:** Fanny Nadia Dissak-Delon, Guy-Roger Kamga, Perrine Claire Humblet, Annie Robert, Jacob Souopgui, Joseph Kamgno, Stephen Mbigha Ghogomu, Isabelle Godin

**Affiliations:** 1 Ministry of Public Health, N°8, Rue 3038 quartier du Lac, Yaounde, Cameroon; 2 Ecole de Santé Publique - Campus Erasme, Université Libre de Bruxelles, Route de Lennik 808 CP591, 1070 Brussels, Belgium; 3 Molecular and Cell Biology Laboratory, Department of Biochemistry and Molecular Biology, University of Buea, P.O. Box 63, Buea, Cameroon; 4 Institut de Recherche Expérimentale et Clinique, Université Catholique de Louvain, Clos Chapelle-aux-champs 30 bte B1.30.13, 1200 Brussels, Belgium; 5 Institute of Molecular Biology and Medicine, Université Libre de Bruxelles, Rue des professeurs Jeener et Brachet 12, Gosselies, 6041 Charleroi, Belgium; 6 Centre for Research on Filariasis and other Tropical Diseases, P.O. Box 5797, Yaounde, Cameroon; 7 Faculty of Medicine and Biomedical Sciences, University of Yaoundé 1, P.O. Box 1364, Yaounde, Cameroon

**Keywords:** Onchocerciasis, Stakeholders experience, Community intervention, Qualitative survey

## Abstract

**Background:**

The global burden of onchocerciasis is the heaviest in sub-Saharan Africa. Studies have shown the importance of the role of Community-Directed Distributors (CDDs) and nurses in onchocerciasis control, but little is known about their experience in implementing onchocerciasis control programmes. Our aim was to document the barriers that CDDs and local health administrators face in implementing onchocerciasis control activities.

**Methods:**

We conducted a qualitative survey consisting of 16 in-depth interviews and 8 focus group discussions (FGDs) across three health districts of Cameroon. We interviewed a total of 9 local health officials at the district and Health Area levels, and 7 CDDs. Eight FGDs were conducted with CDDs and Health Committee members.

**Results:**

The major barriers to the implementation of Community Directed Treatment with Ivermectin that we identified were linked and interrelated. Examples of these barriers included: contextual factors (geographical and cultural background), top-to-bottom planning, insufficient human and material resources, and lack of transparency in the management of the programme’s funds.

**Conclusions:**

The CDTI at operational level still faces many obstacles which negatively affect therapeutic coverages. This can lead to the non-adhesion of the communities to the programme, consequently jeopardizing the sustainability of the onchocerciasis elimination programme. We recommend that the national programme planners put in place a transparent management and planning system for onchocerciasis elimination activities, with better communication with local programme stakeholders.

## Background

Onchocerciasis is a vector-borne parasitic disease caused by the nematode *Onchocerca volvulus* and transmitted to humans by the bite of infected flies belonging to the *Simulium* genus. The consequences of this disease include irreversible blindness, severe itching and disfiguring skin lesions. Besides causing human suffering, the disease also leads to a high socio-economic burden in affected communities. Onchocerciasis mostly occurs in tropical zones, including Latin America (Venezuela, Brazil), Asia (Yemen) and Africa. Sub-Saharan Africa in particular carries the heaviest burden of the disease, as more than 99% of cases are found in 31 African countries [1].

The control and even elimination of onchocerciasis is possible. One of the best examples of onchocerciasis elimination has been observed in Latin America, where a strategy consisting of a biannual large-scale treatment with ivermectin was carried out. According to the World Health Organization (WHO), Guatemala became in 2016 the fourth country in the world after Colombia (2013), Ecuador (2014) and Mexico (2015) to be verified as free of onchocerciasis [1].

From 1995 to 2015, the fight against onchocerciasis in sub-Saharan Africa was spearheaded by the African Programme for Onchocerciasis Control (APOC). The programme’s main strategy was the annual mass distribution of ivermectin, known as the Community Directed Treatment with Ivermectin (CDTI). In 2014, this treatment was received by over 112 billion people across the continent, with more than 65% of therapeutic coverage. As a result, it is estimated that the prevalence of onchocerciasis infection was reduced by 73% compared to pre-APOC levels [2]. After APOC’s mandate closed in 2015, the responsibility of onchocerciasis control was transferred to Ministries of Health with the goal of establishing “*country-led systems capable of eliminating onchocerciasis as a public health problem*” in their respective countries [3].

Despite about 20 years of mass distribution of ivermectin in Cameroon (a country member of the APOC), onchocerciasis transmission still persists [4, 5]. The prevalence of the disease in many CDTI zones in Cameroon remains higher than the predicted values [6, 7], thus defeating the elimination goal set by the country. This can be explained by multiple interrelated determinants, including factors related to humans, parasites, vectors and the environment. Recent studies addressing human factors associated with the elimination of onchocerciasis have emphasised treatment adherence [8–11]. Findings from these studies revealed that beneficiaries’ adherence is mainly influenced by organizational factors such as operational aspects of ivermectin distribution campaigns, or people’s perception of the Community Directed Distributors’ quality of work [10, 11].

A Community-Directed Distributor (CDD) can be assimilated to what Glenton et al. [12] define as a lay health worker, which is a “*person who has received some training to deliver healthcare services but is not a health professional*”. Within the framework of onchocerciasis control/elimination, CDD tasks include: conducting village census to determine the number of ivermectin tablets required, administering ivermectin tablets with respect to dosages and exclusion of contra indicated persons, keeping an inventory of ivermectin, treating minor adverse reactions, referring people with severe adverse reactions to the nearest health facility, keeping records and reporting to the health workers [13]. Note that many health workers on the field as well as researchers have a slightly different definition of the acronym CDD (community drug distributor in their case), but they refer to the same people. In this paper we preferred to align with the same acronym definition found on the WHO/APOC website [13], which is “community directed distributors”. In Cameroon, CDDs work in collaboration with local health administrators (Chiefs of Health Areas/District Medical Officers) with a medical/paramedical background. In the CDTI zones of Cameroon, besides medical care, the staff are also in charge of training CDD, supervising their work on the field, and managing side effects according to the technical platforms of their health facilities.

Along with the above cited articles, the core role played by CDDs and medical personnel in onchocerciasis control in Cameroon was also raised by Njim and Aminde [14], who found that one of the weaknesses of the National Onchocerciasis Control Programme (NOCP) was related to the insufficient number of CDD and the insufficient knowledge that health care professional have about the disease. Many authors have underpinned the importance of CDD and nurses in the control of onchocerciasis and of other neglected tropical diseases, as summarized by Corley et al. [15] who found more than 50 articles on this topic.

Besides the importance of nurses and CDDs in onchocerciasis control, the issue of the factors that influence their motivation to perform their tasks is progressively discussed in the literature. A review published in 2018 [16] found that several cultural, health systems and financial challenges have a significant impact on the motivation of CDDs. In continuity of these authors, in this study we aimed to document the challenges of CDDs and the views of health professionals on onchocerciasis control/elimination programme in Cameroon.

Assessing the experience of these local key actors on onchocerciasis control would be valuable for national onchocerciasis control planners. It will help to adjust strategies to achieve the onchocerciasis elimination goal. The present study therefore aims to document the barriers that CDDs and local health administrators face in implementing onchocerciasis control activities at peripheral level in Cameroon.

## Methods

### Settings

The present study follows a first quantitative survey which aimed at assessing the determinants of beneficiaries’ adherence to ivermectin in 3 rural Health Districts (HDs) in the West, the Centre and the Littoral Regions in Cameroon. These HDs were selected because of the persistence of onchocerciasis transmission [11]. The main findings of that quantitative study showed the importance of factors related to the organisation and implementation of the programme in people’s adherence, namely the CDDs’ quality of work [11]. We therefore set out to assess CDDs’ and local health administrators’ opinion of the organizational bottlenecks of onchocerciasis control at an operational level.

### Context of the operational level of care in Cameroon

The Cameroon health system, usually described as a “health pyramid”, is divided in 3 levels [17, 18]. The top level, or “central level”, is administratively constituted by the Ministry of Public Health’s headquarters. The central level comprises the main directions (direction for family health, direction for disease and epidemics control among others), and the representatives of different health programmes (for instance, NOCP; Extended Programme of Immunization). These entities are responsible for the political leadership of the health system. The middle of the pyramid, or “intermediate level”, is composed of the Regional Delegations of Public Health, with the regional bureaus of the main health programmes. Their role mainly consists of providing technical assistance to HDs.

The base of the pyramid, the “operational level”, is the site of implementation of health policies and strategies. This level is represented by the HD, which in turn comprises several Health Areas. Here, the medical administrative staff work in collaboration with beneficiaries through dialogue structures called Health Area Committees (HACs). Likewise, communities can participate in the management of the public health facilities through Hospital or Health Centre Management Committees (HMCs). The members of both HACs and HMCs are supposed to be elected every two years by their communities to whom they are accountable.

### Study design and participant selection

To better explore stakeholders’ experience with the implementation of onchocerciasis control in their local contexts, we chose a qualitative approach which is “*concerned with discovering the meanings seen by those who are being researched and with understanding their view of the world rather than that of the researchers*.” [19]. Individual interviews and focus group discussions were chosen as research methods.

The field work took place in July, August and December 2016, focusing on one HD per month. In each HD we randomly selected 2 Health Areas (HA), where we conducted individual interviews and focus group discussions (FGD). Interviews were conducted with former CDDs who resigned from their positions during the previous ivermectin distribution campaign to understand the underlying factors that led to their decisions. These former CDDs were identified with the assistance of the Chief of Health Area (CHA) who provided us with 2 to 3 names of ex-CDDs. According to the information gathered during the interviews, we had planned to look for other ex-CDDs when necessary, using the principle of snowball recruitment. We stopped the enrolment of ex-CDDs when we reached saturation. In-depth interviews were also undertaken with the Chiefs of the selected HA and one official at the District level, either the District Medical Officer or the Chief of Bureau Health.

The composition of our focus group discussions (FGD) was done conveniently with the help of the respective CHA and presidents of the Health Committees, who informed and made appointments with eligible participants. Separate groups were formed for active CDDs and HAC members. We also planned separate groups according to gender.

### Data collection

Interviews were performed either at the homes or workplaces of the participants, while FGDs took place in public places such as primary schools, village social halls, or other places selected by the participants. In order to facilitate discussion within the groups, younger participants (less than 25 years-old) were separated from their elders in each focus group. The language used in all the interviews and FGD was French, which is the predominant official language in the 3 regions of our fieldwork (West, Centre and Littoral regions).

Since we adopted an inductive approach, we had no hypothesis before going to the field. Interviews and FGD topic guides were therefore built on general questions to be discussed with the respondents. The main focus of the exchanges was the participants’ experiences with the implementation of the onchocerciasis programme, both the positive and negative aspects. Ex-CDDs were also asked to discuss the circumstances that led them to resign the activity. During the interview of CHA and District Health Service staff, we also proceeded to a first validation of our findings, by giving them a report of the general ideas coming from the field and acknowledging their opinion about the plausibility of our primary conclusions.

### Data analysis

All the interviews and FGD were audio taped, allowing iterative analysis as fieldwork was ongoing. Findings from the preliminary analysis lead to adjustment of probes in subsequent interviews and FGDs.

Audio tapes were transcribed by a research assistant. The accuracy of transcriptions was verified by one author. Data analysis was conducted using a general inductive approach. Thomas [20] defines this analysis approach as a systematic procedure for analysing qualitative data with regards to the study objectives. Using the inductive approach included: raw data organization, repeated thorough reading of all the transcripts, identification of the first emerging categories, and merging/refining of those categories [21]. More specifically in our study, these steps were followed and controlled separately by three of the authors.

In order to preserve respondents’ anonymity, the names of their communities were coded into “villages”, so we had from village 1 to village 6. Then we aligned the names of the corresponding HA, to have “HA village” from 1 to 6. Finally, the HDs were coded with the numbers of their corresponding HA, so we had HD 12 for HA village 1 and 2, HD 34 for villages 3 and 4, and HD 56 for HA villages 5 and 6.

## Results

We carried out individual interviews with a total of 9 local health officials: 3 District Officials (one per HD, either the District Medical Officer or the Chief of Health Bureau) and 6 Chiefs of Health Area (one per HA). The characteristics of each respondent are detailed in Table 1. Concerning community workers, we undertook 7 individual interviews with resigned CDDs (see Table 1) and 8 FGD with CDDs and Health Committee members.

**Table 1:** Characteristics of the participants, individual interviews

Role	Gender	Qualification /profession	No. of years in the CDTI
District official	Male	Nurse	8
District official	Male	Administrator	6
District official	Male	Physician	14
Chief of Health Area	Female	Nurse	3
Chief of Health Area	Female	Nurse	8
Chief of Health Area	Male	Nurse	22
Chief of Health Area	Female	Nursing aid	2
Chief of Health Area	Female	Nurse	2
Chief of Health Area	Male	Nurse	4
Resigned CDD	Female	Farmer	Unknown
Resigned CDD	Male	Pastor	10
Resigned CDD	Male	Trader	2
Resigned CDD	Male	Farmer	10
Resigned CDD	Male	Farmer	4
Resigned CDD	Male	Farmer	15
Resigned CDD	Male	Traditional healer	Unknown

*Abbreviation*: CDTI, community directed treatment with ivermectin

The FGD were composed of an average of 8 participants, with a minimum of 5 and maximum of 12 participants per group (see Table 2 for details). In most of cases women requested mixed groups. In-depth interviews lasted 30 to 51 minutes, while FGDs took an average time of 45 minutes.

**Table 2:** Characteristics of participants, focus group discussions

	No. of participants	Gender distribution	Participants’ roles	No. of years in the CDTI (min–max)
FGD 1	5	5M	Mixed: 3CDD/2HAC	2–20
FGD 2	6	4F/2M	HAC	4–15
FGD 3	5	1F/4M	HAC	Unknown
FGD 4	5	5M	HAC	4 for all
FGD 5	12	10F/2M	CDD	0–4
FGD 6	5	5M	CDD	3–20
FGD 7	7	4F/3M	CDD	2–14
FGD 8	6	3F/3M	CDD	4–16

*Abbreviations:* CDD, community directed distributor; CDTI, community directed treatment with ivermectin; F, female participants; FGD, focus group discussion; HAC, health area committee; M, male participants

The data analysis process allowed us to identify 5 general emerging themes from the individual interviews and FGDs: local health system organisation and responsibilities of the stakeholders, facilitators of NOCP implementation, barriers to NOCP, the influence/relationships between local health officials and the dialogue structures, and lastly, the issue of CDD motivation. Focusing on the barriers to NOCP, we initially had 21 categories that emerged from our data. At the end of the process of reduction of overlapping and/or redundant categories within and outside the “barrier to NOCP” topic, we finally identified 4 major bottlenecks to the implementation of CDTI.

**Fig. 1:**
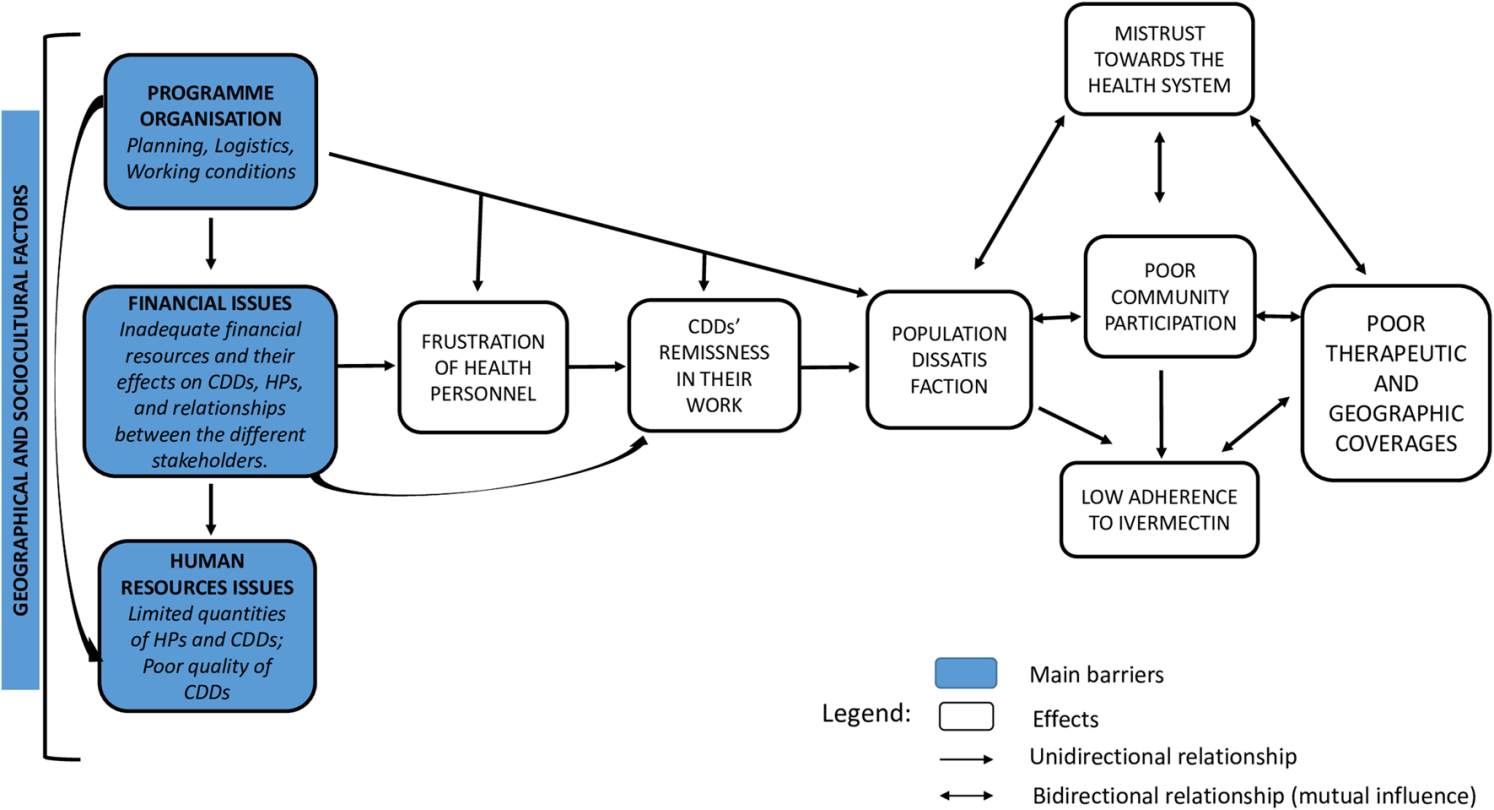
Main barriers to onchocerciasis control at peripheral levels: their inter relations and effects. *Abbreviations*: CDDs, community directed distributors; HPs, health professionals

These bottlenecks were linked and could have mutual influence, as summarized in Fig. 1. They included: contextual factors, programme organization, financial issues and human resources issues.

### Geographical and cultural context

The geographical threat to onchocerciasis elimination activities on the field was mainly observed in the rural areas. Rural settlements in the districts of our study were characterized by low population densities scattered over large areas and have non-passable roads. These 2 main characteristics posed an obstacle during ivermectin distribution to CDDs who distribute the drugs, and to CHA and District Officials (DO) who supervise the distribution.

“*In town it is easy, you divide squares of 50 to 100 houses. But in rural zones a CDD has a linear of 15 to 20* *km. That’s what makes work harder then.*” (Chief of Health Area, Interview, HA village 1).

Socio-cultural barriers in general stemmed from some people’s mistrust of medicine distributed freely to everyone. This led to rumours regarding the quality of ivermectin provided by the programme. We note that doubts on ivermectin quality or efficiency concerned only the drug provided by the programme and not the molecule in general. Indeed, our respondents described that people used to purchase ivermectin from illegal vendors, especially during the period between two campaigns when drugs were not available in the normal drug delivery system.

Other cultural barriers were more specific to semi-rural areas, locally considered as small towns where economic activity is more developed than in neighbouring rural settlements. Semi-rural zones are socially characterized by people coming from different origins, with different cultural backgrounds and who barely interact with each other. This social pattern negatively influences CDDs acceptability in comparison with rural zones where people share the same geographical area and the same ethnic cultural values.

“*Here* [in semi-rural area] *there is a cultural diversity, you find all kind of tribes. Yet for a CDD, being from the same tribe can influence; by speaking the same mother tongue than somebody, he can agree to drink the drugs. Moreover, here in town we don’t really know each other, there is not solidarity in town like in the village!*” (Chief of Health Area, Interview, HA village 5).

### Programme organization

The respondents mentioned some failures in NOCP organization which could become obstacles to the programme’s implementation in the field. These deficiencies mainly concerned planning, logistics and working conditions.

#### Programme planning

Three main insufficiencies around programme planning emerged from our interviews and FGDs, including: wrong period of ivermectin distribution, brief presence in the field, and agenda conflict with other health programmes.

The interviews revealed that the distribution period in the field is determined by the NOCP. Participants complained that ivermectin distribution campaigns were generally organized during school holidays, a period during which the population flow is quite important. This often results in lower therapeutic coverage, because many people are absent during the campaigns.

“*We suggest that CDTI campaigns should be organized in June, while parents and children are still present. When the campaigns are organized in August, my children, my population leaves for holidays and complain at their return that they did not received ivermectin*.” (Chief of Health Area, Interview, HA village 6).

Moreover, these holiday periods coincide with the rainy season. During this time, abundant rains have the effect of accentuating the geographical barrier because the roads leading to the remote areas become even less practicable.

In addition to the wrong period of distribution, our interlocutors deplored the fact that the only activity for onchocerciasis control planned by the NOCP for the operational level was the annual mass distribution of ivermectin.

“*I think that the Programme managers should maintain communication. Oncho programme has a problem of permanent communication. When we launch the activity* [ivermectin distribution]*, we communicate about oncho during one month; as soon as the activity ends, we don’t talk about oncho anymore, unlike the other programmes.*” (Chief of Health Area, Interview, HA village 1).

Another planning issue raised by the District and Health Area officials was a concern over the interference of the other health programmes, marked by conflicts in the execution of various activities in the field during the same calendar period. For instance, DOs and CHAs reported that immunization campaigns used to coincide with ivermectin distribution campaigns.

#### Programme logistics

During the interviews, most of our respondents reported late arrival of drugs and other inputs used during the campaigns (leaflets, dispensing registers, report forms). According to them, drugs are sometimes provided after the beginning of the distribution process, for instance after the CDDs training.

“*It is the District that informs us that Mectizan is available. Sometimes, they say that we will share the drug at the end of the month. But when the end of the month comes they say that we should wait, the Region has not yet sent the drugs*.” (HAC Member, FGD, village 6).

Besides late arrival of ivermectin, some CDDs also reported insufficient drug supply compared to the quantities estimated at their censuses.

#### Working conditions

The planning of ivermectin distribution during the rainy season that we mentioned above also had consequences on working conditions. The CHAs and CDDs mentioned that the effect of this wrong planning was worsened by the absence of adequate equipment provided by the NOCP.

“*CDDs start to get discouraged (…) they asked for rain coats, they asked for boots, they asked umbrellas, it have not been provided*” (Chief of Health Area, Interview, HA village 2).

In parallel, the participants also noted that the work of CDDs on the field is sometimes hampered by the absence of proper identification. They reported for instance that in absence of badges or other proof of their work, they have difficulties in accessing some households. This difficulty is present despite the T-shirts provided by the NOCP.

“*At times the T-shirt is used during the 1st of May parade. Many people wear it, even if they are not CDDs, so it brings confusion. But with badges…, like vitamin A workers, they have signs proving that they are from the health sector*.” (CDD, FGD, village 1).

### Financial aspects

Almost all the stakeholders during interviews and FGDs spontaneously mentioned financial related issues as their main difficulties. DOs and CHAs specifically complained of financial planning of the ivermectin distribution process that was not consistent with their local realities. The most salient effects of this insufficiency of financial resources on NOCP implementation at operational level included: CDD demotivation and resignation, difficulties for local health officials in organizing and supervising ivermectin distribution campaigns, and the deterioration of trust relations between the actors in the field.

#### CDD demotivation

All participants voiced the CDDs’ unhappiness towards the programme’s lack of remuneration. This insufficient remuneration was identified by our respondents to be a major cause of CDD attrition. Moreover, this was also reported to be the main reason why villagers were reluctant to be enrolled as CDDs.

The issue at stake was the nature of CDDs’ voluntary work. The desire to contribute to the well-being of the people of their village is usually perceived to be enough to motivate them. However, the main barrier to CDDs volunteering was the general economic situation. According to participants, the society where they live is becoming more materialistic, therefore people consider it legitimate to claim a salary for any work carried out.

“*Volunteering doesn’t exist… It is not the fact of an ancestral culture, it is the change of mentalities: as time passes, there are mentalities that change. And also, we are in a capitalist country!*” (District Official, Interview, HD 12).

More specifically, CDDs reported the opportunity costs and shortfalls generated by the time spent distributing ivermectin.

Furthermore, CDDs also participate in other health programmes where monetary incentives are more important than that provided by the NOCP, thus increasing the dissatisfaction of CDDs towards the programme.

“*But I also noticed a demotivation of CDDs (…), they say that what they are given as motivation is much lower than other programmes, perhaps as the EPI* [Expanded Programme of Immunization] *(…) Therefore, they feel wronged*” (Chief of Health Area, Interview, HA village 1).

#### Financial difficulties for local health officials

District and Health Area officials revealed that the inadequate financial planning of NOCP at their respective levels was mainly marked by underestimation of travel costs. Indeed, they must travel several times for preparatory meetings before the campaigns and for supervision of the dispensation in the households during the campaigns.

“*Leaving from my community and go to the District headquarters costs 15,000 CFA francs for the round trip. I pay that amount, to attend a meeting where I’ll have a per diem of 1000 francs, it is not easy*”. (Chief of Health Area, Interview, HA village 1).

#### Deterioration of trust relations between the actors

The local health officials revealed that people in their milieu have a general perception of a well-financed health sector in Cameroon. Some of them explained that this perception was reinforced in people’s minds due to regional or national level staff using all-terrain vehicles when travelling to their localities for supervision. This idea of a sector well financed, in the context of limited financial resources, has led to the suspicion of a non-transparent management of the funds. In such a situation, each person assumes that his direct supervisor diverts the funds allocated for the activity.

“*You know that when money crosses many steps, at operational level it cannot be the same like what was planned!*” (Chief of Health Area, Interview, HA village 5).

The District Officials were aware of these rumours, which they spontaneously brought up during the interviews without being prompted. These District Officials seemed to take it inevitably, but in their remarks, we could also detect some discomfort, expressed in “lack of confidence”.

“*If you see the means that are given to cover all this activity, it is really not simple. And what we give to the chiefs of Health Areas is obviously insufficient. This makes that we don’t feel in confidence*.” (District Official, Interview, HD 34).

Finally, we noted that the confidence relationship that exists between community workers and their communities also deteriorated due to the same financial issues. With the progressive withdrawal of APOC, communities are asked to provide incentives to their CDDs. These incentives can be monetary or in kind and can be given either by individuals or through the municipal councils. Nevertheless, according to the health officials and community workers, the community incentives are not easy to obtain because of this very popular perception that the health sector is highly funded.

“*Non-monetary incentive, that is for example people helping CDDs in their housework, is feasible. But it is not easy to execute in practice. Even in our remote villages where the social cohesion is high, when you talk about helping CDDs in their farms, villagers say that they cannot work for people who work and receive salaries. It is difficult in rural communities, and almost impossible in urban communities*.” (District Official, Interview, HD 56).

### Human resources aspects

#### Limited quantity of health professionals and CDDs

All the CHAs noted that in their responsibility zones, their work was more difficult because of the insufficient number of collaborators.

“*You train somebody and work with the person, then the person leaves when he has an opportunity in town. So you remain alone with all that work. People refuse to come work in remote areas*” (Chief of Health Area, Interview, HA village 2).

The insufficiency of health personnel posed an obstacle to the supervision of the ivermectin distribution campaigns. It also hindered adequate healthcare offered to the population. Most of the CHAs were also physicians/nurses with responsibilities at the hospital/health centre. At times, some of them were the only health professional at their facility. Consequently, they reported some conflicts between their curative activities at the hospital and public health duties in the field. Repeated absences because of public health activities were, according to them, a source of patient dissatisfaction, which could rupture the confidence of the populations towards the health system in general.

The limited human resources was also a problem among the CDDs. The first reason for low numbers of CDDs was due to the NOCP planning not following the needs expressed by the peripheral level health officials.

“*Now the problem of oncho, is the CDDs. The number of CDDs that they give us is very insufficient! I consulted the archives and saw that last year they gave 520 CDDs. This year, they authorized only 500 CDDs, whereas the 520 of last year was not enough*!” (District Official, Interview, HD 56).

In addition to the inadequate CDD plan execution, the number of CDDs declined in communities as a result of a progressive demotivation of villagers and difficulties in recruitment.

#### Poor quality of CDDs

The CHAs and experienced CDDs complained that some of the few active volunteers often tend not to take their task seriously. This lack of seriousness is marked by distraction during the training, lack of communication with beneficiaries, low coverage of their work areas (especially the most remote areas), and inadequate ivermectin administration by some CDDs.

This scarcity of volunteers has also been reported by CHAs as one of the reasons that have led them to make less rigorous decisions in the selection of CDDs. The first criterion in which they became less rigid pertains to the CDDs working zones. Under normal circumstances, CDDs are supposed to work in the community where they live and are known by the inhabitants, to facilitate the acceptability of their role. However, in practice, this is not always the case.

In addition to the risk of non-acceptability, the non-membership of the CDD in a community jeopardises the geographical coverage of the campaign, especially for revisits.

“*Especially me, I have to pay transport to reach there. So when I arrive, if I don’t find anyone I’ll not return there because I pay transport to go there*” (CDD, FGD, village 3).

Another essential criterion in which the CHAs have become less observant due to the scarcity of volunteers, concerns the communication and writing skills. Overlooking these skills has also hampered the ongoing and reporting of ivermectin distribution campaigns:

“*We have serious problems. CDDs selection is hard because the people we are going to take in the community do not even master anything! (…) That is to say: firstly, to express oneself, and secondly to be able to write French, to be able to fill a register*.” (District Official, Interview, HD 34).

## Discussion

The present paper uncovers emerging themes relating to Cameroon’s capacity to achieve their onchocerciasis elimination goals, particularly in the context where after 20 years of control, onchocerciasis transmission is still prevalent in many areas of the country. The aim of this study was to understand operational stakeholders’ perceptions of the local barriers that hinder the implementation of CDTI in the field. Recent studies investigating this issue in the specific context of Cameroon have been based their study in the South West region of the country, where onchocerciasis transmission is still ongoing [22]. Building on these previous studies, we explored the realities experienced by stakeholders in the West, the Centre and the Littoral regions, where the prevalence of onchocerciasis is also above the expected values [7, 23].

Inductive analysis of our data allowed us to identify three main factors directly associated with the NOCP which constitute obstacles to its success. These are: programme organization, lack of adequate human resources and insufficient financial resources. Besides these factors, we also identified geographical and cultural factors that constituted external barriers to NOCP implementation on the field. They are important to take in consideration when analysing the NOCP system at the operational level, because of their interactions with the inner barriers of the programme.

We found that the consequences of a limited human workforce on the NOCP were exacerbated when accounting for the geographical context. The remoteness of CDTI zones and the hard conditions of life discourage health personnel from joining and remaining in those settings. In a strategic briefing note written in 2011, Mba et al. [24] pointed out that the main factors leading to health professional’s departure from remote areas included the absence of compensatory measures for the high cost of living, isolation from family and the lack of a clearly defined career path. The minority of health personnel who agreed to stay in the CDTI zones, despite a lack of interest in working in remote places, would often complain about the workload, which became a source of frustration. Work overload and work-related frustrations are documented causes of poor professional commitment and burnout among the health personnel [25–27]. In the specific framework of onchocerciasis control, poor professional commitment among health personnel can result in poor supervision of the CDD activities and poor accompaniment of the community. The importance of the CDDs supervision by health professional and its significant impact on treatment coverage has been demonstrated by Katabarwa et al. [28] in a study conducted in Cameroon and Uganda.

Inadequate programme planning was identified as a limiting factor of NOCP implementation at the operational level. The community has practically no control on the period of distribution, which is a very important part of the ivermectin distribution campaigns. For instance, we found that the campaigns usually take place during the rainy season which creates unfavourable working conditions for CDDs and health professionals, and thus lowers geographical and therapeutic coverages. Likewise, in recent studies, Kamga et al. [29] and Duamor et al. [22] also found that in various CDTI zones in Cameroon, the community does not have a say on the period in which ivermectin is distributed, resulting in low therapeutic coverage and poor community appropriation. Another major threat to expanding therapeutic coverage in accordance NOCP planning, was the insufficient and at times delayed drug supply at the community level.

Financial issues were reported by respondents to be one of the most critical barriers to NOCP implementation at the operational level. At first sight one could think that the only problem was the amount of money dedicated to the programme. However, our findings revealed 3 major finance-related issues that really threatened the sustainability of the onchocerciasis programme at the operational level.

The first issue pertained to the role of CDDs, whether it should be a volunteer *versus* a remunerated position. In theory, CDD work is voluntary and official reports dating back ten years and more have indicated that CDDs’ motivation was more of non-monetary in nature [13, 30]. However, our findings revealed that the lack of monetary incentive was a major cause of attrition among CDDs, and a discouraging factor for other community members to enlist as CDDs. Deeper analysis of our respondents’ statements showed that what people commonly called “incentives”, was comparable to a salary, and that was the reason why they found the given amounts to be inadequate for the job that they perform. Consistent with our findings, recent authors working on onchocerciasis programmes or other programmes using a Community Directed Intervention (CDI) approach, such as lymphatic filariasis or schistosomiasis, found that the absence or insufficiency of financial incentives was a challenge for these programmes [22, 31–33]. These differences in authors’ findings after ten years can be explained by a change in mentalities, due to socio-economic realities and the impact of globalization. Indeed, with the advent of new information and communication technologies, especially access to the internet, people in rural or semi-urban areas are more aware of the contemporary world around them. Consequently, the programme should consider how populations perceive the monetarisation of many things in their environment, making it more difficult to support volunteering.

The second financial related issue was the financial planning of the programme. This second issue is one of consequences, as historically Cameroon has had difficulties setting up its own sustainable financing strategy of CDTI in the specific framework of onchocerciasis control. As reported by Meredith et al. [34], implementing CDTI in Cameroon was challenging because the country decided to align the strategy in the cost recovery approach that was applied for health in general. However, due to lower results than what was observed in other countries, international pressure has led the country to finally agree to make ivermectin free for beneficiaries [28, 34]. Nevertheless, the question of CDD incentives remains unsolved, and unfortunately the government’s decision to pay the CDDs ended in failure, since the payments were irregular [22, 34]. This reported irregularity of the payments from the government can explain the mistrust of CDDs that we encountered towards the Health Areas and District Officials, who were suspected of misappropriating the funds they owed. Since 2013, the responsibility of providing incentives to CDDs has been transferred to the communities. However, the transition to this new system was not formally communicated to communities. Consequently, communities were suspicious when asked to pay for a drug that they knew was free. In the context of health financing and health aid, African governments are generally aware of the necessity of transparency towards donors in order to maintain partnerships [35]. In the same vein, transparency is also needed towards populations in order to avoid mistrust between health workers and beneficiaries. For instance, we observed that the NOCP usually provides posters and pamphlets to advertise about the dangers of onchocerciasis and lymphatic filariasis, and the necessity of taking ivermectin and albendazole during campaigns. These same posters, added to official radio and television advertisements, could be used by the NOCP to inform populations about the system of allocating incentives to CDDs.

The third financial related threat to the NOCP at the operational level was related to the general economic context of the country. Okalla and Le Vigouroux [17] described that in practice, a District Medical Officer will never receive the correct amount allocated by the government to his district because of “tips” and other formal or informal procedural costs previously spent to get in possession of his budget. This reality is well known by the Chiefs of Health Areas and even some community leaders, which could explain why they barely trust the amounts they are provided during an activity.

Inadequate CDD planning by NOCP, hard working conditions and poor remuneration were the main reasons for the decline in CDDs on the field. Many CDDs progressively resigned as a result of these factors, and villagers became less interested in the role. Consequently, the scarcity of volunteers was mentioned by CHAs as a reason to be less strict about the skills required for CDDs, such as possessing communication and writing skills. Writing skills can be included in the WHO’s “good literacy” qualities of CDDs [13], and are important for CDDs because they need to report on their activities at the end of the drug administration campaign. The reports produced by CDDs are valuable because they constitute the basic unit of the general report of the NOCP. The data used by the NOCP to monitor programme achievements, plan activities and inform international partners, come from reports generated by CDDs in their communities. These reports are then compiled level by level (HA – HD – region) until they reach the national level. If CDDs write incorrect reports, this could raise doubts about the quality and reliability of the reports used and shared by the NOCP. Such reporting errors could explain why Kamga et al. [29] found survey coverages in the same regions as where our study was conducted to be significantly lower than the reported coverages, with differences up to 22%.

Furthermore, our findings show the importance of considering contextual and programme organizational factors in the implementation of a community-based intervention. Beyond the specific context of onchocerciasis, the barriers to NOCP reported by our may also affect other community directed interventions. By definition, the Community Directed Intervention (CDI) is “*an approach in which communities themselves direct the planning and implementation of intervention delivery*” [30]. This method is described to bring about better results in terms of community ownership and empowerment concerning their health [30, 34]. However, our results revealed that in practice, communities are not actively involved in the organization and planning of health activities and have no clear idea of the financial management of the programme activities. Such conditions promote the non-adhesion of the communities to the programme, thus jeopardizing the sustainability of the CDTI approach in general.

### Study limitations

One of the main criticisms often addressed against qualitative research is that the results cannot be generalized [36]. To minimize this limitation and enhance the quality of the study, we have accounted for different criteria such as credibility (respondents triangulation, investigators triangulation, member checking) and transferability (resonance with existing literature) [36, 37]. As it pertains to triangulation, study respondents came from three different regions of the country, and in each HD we recruited from two different Health Areas. We also ensured variability in age, experience and gender in the choice of the CDDs and HAC members. Furthermore, we applied the principle of interrater reliability, in which raw data were independently reviewed by different authors. The results were afterwards compared and discussed together. Concerning transferability, we think that the fact that our main findings are consistent with contemporary authors from various countries and within different health programmes that use the CDI approach, is a good indicator of the accuracy of our results.

## Conclusions

In the framework of the onchocerciasis elimination programme in Africa, former APOC countries including Cameroon have decided to scale up the CDTI approach. However, the implementation of this strategy still faces contextual barriers (geographical and cultural) and programme related barriers. Top-to-bottom planning of ivermectin distribution campaigns can affect therapeutic coverage because of difficult working conditions for health professionals and community health workers, and because of the absence of the population at the time of distribution. Insufficient human and material resources are also identified obstacles to the proper organization and follow up of NOCP at operational level. Finally, lack of transparency in the management of the funds allocated to onchocerciasis control activities can create a climate of mistrust especially by the population. This mistrust hinders the community appropriation of the elimination of onchocerciasis in their milieu, which in the long term can be a threat to the sustainability of the programme. We therefore recommend that the government: (i) identify and implement a comprehensive policy that encourages health professionals to work in remote areas; and (ii) establish a transparent funding system for onchocerciasis elimination activities, with better communication with the programme stakeholders, especially the community workers. To maintain the intrinsic motivation of CDDs to continue the work, the NOCP planners could also take inspiration from other health programmes in the country like the HIV programme or the Performance Based Financing project in which community workers, recruited on volunteer basis, have contracts and formal salaries discussed during enlistment.

## Data Availability

The datasets used and/or analysed during the present study are available from the corresponding author upon reasonable request.

## Abbreviations

APOC: African Programme for Onchocerciasis Control
CDD: Community-Directed Distributor
CDI: community directed intervention
CDTI: Community Directed Treatment with Ivermectin
CHA: Chief of Health Area
DO: District Official
FGD: focus group discussion
HA: Health Area
HAC: Health Area Committee
HD: Health District
HIV: human immunodeficiency virus
HMC: Hospital (or Health Centre) Management Committee
NOCP: National Onchocerciasis Control Programme
WHO: World Health Organization
